# Hegonon, a New Silver Proteid Compound of Schering

**Published:** 1910-12

**Authors:** 


**Hegonon, a New Silver Proteid Compound of Schering**

Professor Klingmueller of the Dermatological Clinic, University of Kiel, Germany, reports (*Muenchener Medizinische Wochenschrift,* 1910, No. 32) the results of his comparative clinical experiments with hegonon and other silver proteid compounds which he had so far considered the best. The new compound, which is obtained by treating albumose with an ammoniacal solution of silver nitrate, is a light brown powder, readily soluble in water and containing approximately 7 per cent. of organically combined silver. Its aqueous solutions do not coagulate albumen, even on heating and are not precipitated by sodium chloride. The indications of hegonon are the same as those of other silver proteids. So far it has only been systematically tested in gonorrhea, in which one-quarter per cent. solutions have yielded excellent results, recourse to higher concentrations appearing unnecessary. In this concentration the solutions are almost colorless and cause practically no staining.

Professor Klingmueller finds that in the treatment of simple anterior gonorrheal urethritis, hegonon is at least the equal of the other preparations he tested. Its great superiority over other silver proteids, however, lies in the fact that it far more effectively inhibits complications. Of thirty-eight cases treated with hegonon not a single one developed complications (posterior urethritis, prostatitis, epididymitis). The author also finds that complicated cases of gonorrhea, originally treated with hegonon, show a prompter disappearance of the organism than those in which the other preparations were used. He concludes from his experiments that hegonon deserves first place among modern silver proteid compounds.

